# Avatar Therapy for Persistent Auditory Verbal Hallucinations in an Ultra-Resistant Schizophrenia Patient: A Case Report

**DOI:** 10.3389/fpsyt.2018.00131

**Published:** 2018-04-16

**Authors:** Laura Dellazizzo, Stéphane Potvin, Kingsada Phraxayavong, Pierre Lalonde, Alexandre Dumais

**Affiliations:** ^1^Centre de recherche de l’Institut Universitaire en Santé Mentale de Montréal, Montreal, QC, Canada; ^2^Department of Psychiatry and Addictology, Faculty of Medicine, Université de Montréal, Montreal, QC, Canada; ^3^Institut Philippe-Pinel de Montréal, Montreal, QC, Canada; ^4^Services et Recherches Psychiatriques AD, Montreal, QC, Canada

**Keywords:** treatment-resistant schizophrenia, auditory verbal hallucinations, psychological treatment, cognitive-behavioral therapy, avatar therapy, virtual reality, case report

## Abstract

Effective treatment strategies for schizophrenia remain very challenging and many treatment-resistant patients will suffer from persistent auditory verbal hallucinations (AVH). While clozapine is the gold-standard medication for this complex population, many will not respond to this molecule. For these ultra-resistant patients, limited options are available. Cognitive-behavioral therapy (CBT) is the most widely used psychological intervention, though it offers modest effects. With the interpersonal dimension of AVH being recognized, Avatar Therapy (AT), a novel experiential treatment enabling patients to create an avatar of their persecutor and allowing them to gain control over their symptoms, was developed and tested. Results have shown significant improvements in AVH symptomatology. This paper details a case report showcasing the beneficial results of AT for even the most severe and symptomatic cases of schizophrenia. Mr. Smith has been afflicted with the persistency of all his voices for almost 20 years. To our knowledge, this patient tried almost all possible treatments with little efficacy. This case highlights the difficulty of finding an adequate treatment for ultra-resistant patients. Mr. Smith first followed CBT before initiating AT. With AT, he significantly improved in a way that was not observed with any other intervention and these improvements remained afterward. The severity of his positive symptoms as well as his depressive symptoms diminished, and his most distressing persecutory voice disappeared. He was able to regain a life. The effects of AT went well beyond the patient, the morale of the entire family improved. This ultra-resistant case suggests that AT may be a promising intervention for refractory AVH in schizophrenia.

## Introduction

Schizophrenia, a chronic and severe psychiatric disorder, poses significant challenges. Notwithstanding evidence demonstrating the efficacy of antipsychotic drugs for its treatment (1), there is a high variability in treatment response. Up to 30% may be treatment-resistant (TRS) and suffer from persistent psychotic symptoms, notably auditory verbal hallucinations (AVH) (2–4). AVH are hallmark symptoms of schizophrenia (5) as their prevalence may reach close to 60% (6). These symptoms have been associated with adverse outcomes (i.e., substance use disorders, suicidal ideations, lower quality of life/functioning, higher rates of hospitalization) (7).

Clozapine is the gold-standard evidence-based treatment of TRS due to its superiority over other antipsychotic drugs (8). Still, approximately 40 to 60% patients, termed “ultra-resistant patients,” do not have an adequate response. After clozapine, limited pharmacological options are available for those with residual symptoms (9). Switching antipsychotic medication, dose augmentation and several add-on strategies have been employed (10). Unfortunately, none of these strategies have proven to improve outcomes in ultra-resistant patients and guidelines recommend antipsychotic monotherapy (10–13).

Cognitive-behavioral therapy (CBT) remains the most commonly used psychological intervention. According to the cognitive model of AVH, the basis of cognitive-behavioral therapy for psychosis (CBTp) (14), it is not the voice nor its contents that causes anxiety, but rather the way the patient evaluates it. The aim of most CBTp is to reduce distress and increase tolerance to the voice by primarily modifying voice appraisal (15). CBT has shown low to moderate effect sizes. The nature of voice-hearing is heterogeneous and the likelihood of differing subtypes may contribute to this limited efficacy (16). Though, few CBTp trials have targeted AVH in schizophrenia patients responding to well-defined treatment resistance criteria (17). There is, therefore, a need to improve psychological therapies for TRS.

The interpersonal dimension of AVH is increasingly acknowledged (18). Encouraging patients to enter in a dialog with their voices may help them develop more constructive relationships and reduce distress (19). However, establishing communication with an invisible entity is difficult for both patient and therapist. To overcome this, Leff et al. (20) tested in their pilot trial a computerized system enabling patients to create an avatar of their persecutor and allow them to gain control over their symptoms. Their results have been extended in a larger randomized controlled trial (RCT) comparing Avatar Therapy (AT) to supportive counseling (21). Results showed greater effects of the AT on AVH. Our research team independently adapted AT using immersive virtual reality (VR) and compared, in a pilot RCT, AT to treatment-as-usual in TRS, with half consisting of ultra-resistant patients (22). We found significant improvements in AVH severity, as well as beliefs of malevolence, depressive symptoms, and quality of life lasting at the 3-month follow-up. While promising, further head-to-head RCT involving larger samples comparing AT to other proven psychological interventions are warranted. In our next trial, patients were allocated to CBTp or AT. Those who followed CBTp and whose persistent symptoms did not improve could continue with AT if desired.

Here, we report the case story of an ultra-resistant schizophrenia patient, Mr. Smith,^1^ who failed to respond to several antipsychotic treatments, repetitive transcranial magnetic stimulation (rTMS), and electroconvulsive therapy (ECT) before participating in our trial. Also, the patient completed our CBTp before being offered AT. The latter has shown the most positive effects.

## Case Illustration

### History of the Patient

Mr. Smith, a single and unemployed Caucasian male in his early thirties living with his parents, has lived with tenacious AVH for almost 20 years and has attempted a variety of treatments to alleviate his symptoms with poor outcomes.

Mr. Smith has had a burdensome life path. At 14 years old, he started to experiment various substances, including cannabis, alcohol, and psilocybin. This became a problematic pattern precipitating to psychotic states, which led him to many psychiatric hospitalizations. Though, he continued to consume more substances (i.e., cocaine), altering his global functioning; he dropped out of high school after 10 years due to persecutory ideas. He was first diagnosed with substance-induced psychosis, which turned into a schizoaffective disorder. At 17 years old, he was followed by a specialized clinic for the treatment of young adults with psychosis. An antipsychotic treatment (trials of olanzapine then risperidone) was started with slight improvement in symptomatology. His illness continued to develop into adulthood; he was then diagnosed with paranoid schizophrenia.

His delusional and hallucinatory symptoms had a considerable effect on his life and prevented him to do the simplest of activities (i.e., reading the newspaper). Approximately 15 voices were heard every day, and nearly all-day long. They originated from an array of contexts: the army, supreme court judges, serial killers, Jesus, and known actors. The voices were all linked to one another and intertwined in different “scenes” in his head (i.e., “scenes” of war). Moreover, he thought he had supernatural powers. Most of his voices were pejorative; they would insult him, torment him, and give him orders to kill himself.

His voices brought forward auto and hetero-aggressive behaviors. He attempted suicide eight times by medication intoxication as well as arm and neck lacerations. Some attempts were serious and necessitated medical intensive care unit stays. Moreover, Mr. Smith was aggressive toward his family and the treating staff: he beat a staff member during a hospitalization, he fought with his brother and shook his mother.

Mr. Smith’s evolution was marked by eight hospitalizations in the context of disorganization and suicidal behavior, with one hospitalization at the age of 22 lasting almost 4 years, where he benefited from an intensive program in occupational therapy for those with severe psychotic disorders. It consisted of medication adjustments, psychoeducation, and rehabilitation services. All antipsychotics present in the market have been tried (Table 1) with little success except for clozapine. Most were ceased due to their lack of efficacy or due to the occurrence of adverse effects (e.g., morbid obesity, hypertension, and dyslipidemia). Clozapine was introduced after the combination of risperidone-olanzapine that led to little improvements. With clozapine and lithium, his psychotic symptoms were slightly controlled (i.e., his thoughts were less disorganized, his concentration improved). Unfortunately, he developed a severe and intolerable obsessive–compulsive disorder that was unable to be controlled. After 2 years of treatment with clozapine, he developed leukopenia, which led to its discontinuation. Additionally, Mr. Smith tried a variety of non-pharmacological therapies (Table 2). In total, he had 39 ECT treatments providing him little effect. Mr. Smith followed 27 rTMS treatments, though the effect was also minimal. After trying these interventions, as of 2014, his treatment included an antipsychotic not yet marketed in Canada, amisulpride, with risperidone, which lightly to moderately controlled his positive symptoms. With Mr. Smith’s more stabilized psychotic symptoms, while remaining problematic, his treating psychiatrist (PL) believed that he would be more receptive to a novel psychological approach.

**Table 1:** Pharmacological trials.

Medication	Maximum daily dose	Trial duration	Reason/comments
**Antipsychotics**			

HALOPERIDOL	5 mg	(1) 5 months in 2006 (2) 2 months in 2006	(1) Replaced by **Loxapine** (2) In conjunction with **Olanzapine**

PERPHENAZINE	32 mg	(1) April 2006: short 1-week trial (unknown dosage) (2) December 2006: few weeks trial (unknown dosage) (3) August 2007:4 months trial (32 mg/day)	(1) No details (2) In replacement of **Haloperidol** (3) In replacement of **Risperidone**

LOXAPINE	50 mg75 mg200 mg	(1) August to November 2006 (2) July to December 2008 (3) 10 months in 2013	(1) Replaced by **Haloperidol** (2) In conjunction with **Trifluoperazine** (45 mg/day) caused akathisia (3) Stopped because of increased appetite

FLUPENTIXOL	6 mg	January 2007; stopped in April 2007	In replacement of **Perphenazine**

THIORIDAZINE	250 mg	January 24 to June 28, 2007	In conjunction with **Olanzapine**; replaced by **Risperidone**

TRIFLUOPERAZINE	50 mg	March 26, 2008 to March 19, 2009	Stopped because of Akathisia and lack of effectiveness

RISPERIDONE	3 mg4 mg8 mg3 mg	(1) April 1999 to April 2004 (2) June to August 2007 (3) 20 months from 2011 to 2013 (4) Since April 2014	(1) By itself or in conjunction with **Olanzapine**; replaced by **Clozapine** (2) In replacement of **Thioridazine**; in conjunction with **Olanzapine** (3) Stopped because of massive weight gain (reached 300 lbs) (4) In conjunction with **Amisulpride**: added because **amisulpride** was not efficient enough

OLANZAPINE	20 mg15 mg40 mg	(1) January 2001 to April 2004 (2) November 2006 to January 2007 (3) April 2007 to January 2008	(1) By itself or in conjunction with **Risperidone**; replaced by **Clozapine** (2) Restarted after termination of **Clozapine**; replaced by **Thioridazine** in conjunction with **Flupentixol** (3) In conjunction alternately with **Thioridazine**, **Risperidone**, **Perphenazine**; stopped in January 2008 to start a series of ECT

CLOZAPINE	350 mg	April 2004 to November 2006	Introduced because of the little improvement under **Risperidone** and **Olanzapine**. Stopped on November 2006 because of leukopenia; emergence of a severe obsessive–compulsive disorder (OCD) under **Clozapine**

QUIETAPINE	600 mg	Trial in March 2009 (duration of trial unknown)	No details

AMISULPRIDE	1,200 mg	Since January 2014	In replacement of **Risperidone** because of lack of efficacy

ASENAPINE	Unspecified	5 months in 2012	Stopped because of lack of efficacy and irritability

ZIPRASIDONE	100 mg	11 months in 2012	Stopped because of lack of efficacy

ARIPIPRAZOLE	30 mg	Started in 2011	In conjunction with **Risperidone**

**Antidepressants**			

CITALOPRAM	80 mg	October 2004 to April 2007	Introduced after the emergence of OCD under **Clozapine**, stopped after the improvement of the OCD symptoms; mixed effects

FLUOXETINE	80 mg	November 2007 à Mars 2009	Reappearance of the OCD symptoms under **Olanzapine**, stopped because of disinhibiting effect; mixed effects

CLOMIPRAMINE	250 mg	March 2008 to March 2009	In replacement of **Fluoxetine**; mixed effects

**Mood stabilizers**			

LITHIUM	1,800 mg1,050 mg2,400 mg	(1) January 2001 to March 2007 (2) February to July 2008 (3) February 2009 to unknown (possibly March 2009)	(1) Hypothesis of a schizoaffective disorder; associated with **Olanzapine** and then with **Clozapine**. Dosage reduced to 600 mg/day in January 2007. Replaced by **Gabapentin** (2) In conjunction with **Valproate**, retaken because of previous good response (3) Inflated mood and behavior, serum lithium levels remained low

VALPROATE	1,250 mg1,000 mg	(1) April to November 2007 (2) February to May 2008	(1) In conjunction with **Lithium**; improvement of clinical portrait; patient is calmer. Stopped for ECT treatment (2) Restarted because of worsen state; stopped when the patient became stable
GABAPENTIN	600 mg1800 mg	(1) April to June 2007 (2) 4-months trial between July and November 2008	(1) In replacement of **Lithium** (2) In replacement of **Valproate** and **Lithium**; disappointing results

*Bold font represents medication names and total score of the scale*.

**Table 2:** Electroconvulsive therapy (ECT) and repetitive Transcranial magnetic stimulation (rTMS) sessions.

When	Session	Reason/comments
November 2007	Consultation for ECT	The patient came because of an outburst of psychotic symptoms, multiple therapeutic and pharmacological trials were done with no success
December 2007	Series of 14 ECT between December 2007 and January 2008	Calmer, less obsessive–compulsive disorder (OCD) symptoms, lessened voices
February 2008	Maintenance ECT	3×/week, then 1×/week
March 2008	Maintenance ECT	1×/week
March 2008	Maintenance ECT stopped	Large improvement, lessened voices
November 2008	Consultation: he thought about starting a second series of ECT	He punched an attendant, heard the voices constantly except when sleeping. Since the first series of ECT had a positive impact, restarting the ECT was considered
November 2008	Started ECT series, five sessions between November 24 and December 3	Started following a suicide attempt
December 2008	ECT stopped	The patient said he had memory loss and difficulty in concentrating due to the ECT. The Dr. hypothesized that the patient might have Asperger Syndrome, ECT was ended to confirm hypothesis
March 2011	Series of seven ECT	Psychotic symptoms were still very present, changes in medication did not have any success. After the treatment, he said the voices were softer, but still present
April 2011	Maintenance ECT (17 between March and April)	Little improvement, but he said that ECT made him forget his voices
May 2011	Consultation for rTMS	The patient showed interest for a rTMS trial
August 2012	Treatment of 24 rTMS	He still heard the voices, while he slept a little better and had a soothing effect on his anxiety
August 2013	Started rTMS sessions 1×/week	He slept better, had less hallucinatory symptoms, and voices seemed further away
November 2013	rTMS stopped	Dr. said it would not be necessary anymore
Mai 2014	Consultation: he wanted to restart ECT	The patient said he wanted to restart to forget about his voices. The Dr. had doubts as it was not effective in the past

### Course of the Therapies

Mr. Smith was thus referred to our VR clinic. Upon our initial interview, the patient was severely symptomatic and expressed a multitude of delusions. Clinical assessments administered by a research nurse at baseline, after the therapies (CBTp and AT) and at 3 months following AT, consisted of AVH severity, illness symptomatology as well as depressive symptoms, measured with the Psychotic Symptoms Rating Scale (PSYRATS) (23, 24), the Positive and Negative Syndrome Scale (PANSS) (25), and the Beck Depression Inventory-II (26).

#### Cognitive-Behavioral Therapy for Psychosis

Mr. Smith was offered 9-weekly CBTp sessions of 1 h, which was administered in an individual format by an experienced psychotherapist. While he was receptive and attentive, it was recommended to adjust the therapy with shorter sessions.

The sessions involved of a succession of learning modules and task assignments. The first contact consisted in a history of his voices for goal setting. Session 2 and 3 focused on assessing and learning about hallucinations. Mr. Smith completed assignments to reflect on his positive symptoms and the associated triggers. The following sessions focused on metacognition to learn about problem-solving strategies and mechanisms of attribution. Sessions 5 and 6 aided to interpret situations in a better manner and included an assignment to detect the beliefs that were the cause of his ill-being. In sessions 7 and 8, he practiced mindfulness exercises. Session 9 led to the end of the intervention aiming at the prevention of relapse.

During CBTp, there were no improvement in positive psychotic symptoms, and slight reductions of 29 and 7% on the negative and general symptomatology subscales of the PANSS, respectively (Table 3).

**Table 3:** Changes in psychiatric symptoms following cognitive-behavioral therapy for psychosis (CBTp) and avatar therapy (AT).

		Baseline	After CBTp	After AT	Follow-up
PSYRATS		**29**	**29**	**30**	**30**
	Distress	10	9	12	10
	Frequency	9	10	10	11
	Attribution	7	7	4	7
	Loudness	3	3	4	2
PANSS		**112**	**100**	**85**	**84**
	Positive symptoms	30	30	22	25
	Negative symptoms	28	20	20	22
	General symptoms	54	50	43	37
BDI-II	–	**32**	**33**	**8**	**12**

*PSYRATS, psychotic symptoms rating scale; PANSS, positive and negative syndrome scale; BDI-II, Beck Depression Inventory-II*.

*Bold font represents medication names and total score of the scale*.

#### Avatar Therapy

Due to this lack of efficacy, Mr. Smith chose to undergo 7-weekly sessions of AT (one avatar creation session and six 45-min therapeutic sessions).

##### Avatar Selection Phase

Mr. Smith was requested to select the most distressing voice for the creation of the avatar, i.e., *The Judge*. With the support of an experienced psychiatrist (AD), Mr. Smith created an avatar best resembling *The Judge*, which was designed to closely have both the face and the voice of his “persecutor.” The avatar’s face was created using Morph3D character system and the *BehaVR* software and was rendered with Unity game engine. The avatar’s voice was simulated by modifying in real-time the psychiatrist’s voice with the voice transformer Roland AIRA VT-3. Prosody lips synchronization to increase the feeling of presence was performed *via* the SALSA with *RandomEyes* Unity asset. Mr. Smith was immersed in VR through the Samsung GearVR head-mounted display. The virtual environment consisted of an avatar standing in the dark, seen from a first-person perspective.

##### Therapy Sessions

The therapy was provided by a psychiatrist (AD). Prior to the first session, Mr. Smith was asked to write down sentences used by his “persecutor.” Unexpectedly, Mr. Smith provided the psychiatrist with an agenda including these sentences (mostly incomprehensible) with other disorganized details about his “scenes.”

While it was a challenge, the psychiatrist induced a dialog between Mr. Smith and his avatar. In sessions 1–3, Mr. Smith was confronted to the reproduced hallucinatory experience. The psychiatrist would say precisely what the patient wrote or explained, revolving around him being kidnapped, brought to Court, having stopped an atomic bomb and having powers. While the first few minutes of the session created anxiety (7 on a score of 10), he wanted to continue as he was really motivated to remove the main voices (“characters”) of his “scenes” as they were unbearable. Mr. Smith was encouraged to enter in a dialog with the avatar to improve emotional regulation and assertiveness. One of Mr. Smith’s goal was to tell *The Judge* to stop persecuting him and to leave him alone. He was prompted by the psychiatrist to discuss this with his avatar. Self-esteem was the therapeutic target beginning at session 4, which was reinforced by enabling the patients to express himself and to be aware of his qualities. The psychiatrist introduced into the avatar’s dialog a list of qualities provided by his mother (e.g., he is a soft, friendly, and a positive person), father (e.g., he is respectful and appreciated by others), and brother (e.g., he is very resilient). Mr. Smith found it pleasing to obtain such nice thoughts. The avatar progressively became under the Mr. Smith’s control. To do so, the psychiatrist gradually modified the avatar’s speech and tone from being abusive to being more helpful to resonate with the Mr. Smith’s improved ability to regulate his emotions. *The Judge* would now start to tell or question him on his qualities and good things in his life enabling him to become aware of them. For instance, *The Judge* explained to Mr. Smith that his family loved him. Mr. Smith said that he was resilient and learned to control his anger toward his voices. Even though *The Judge* tried to re-irritate him by calling him a thief, he remained firm that he was a decent person. He even told the voice to go away with all the other voices to regain control over his life. In the consolidation sessions, Mr. Smith was encouraged to apply what he had previously learned. Also, as *The Judge’s* voice and the “scenes” decreased, he had the opportunity to highlight these improvements.

At the beginning and the end of each AT session, the psychiatrist discussed with Mr. Smith to measure his general evolution. Early sessions were difficult both for Mr. Smith and AD who had to disentangle the hallucinatory experience. While the prognosis was initially poor, changes were observed early on. Throughout the sessions, Mr. Smith began to accept that the voices and related “scenes” were false and part of his illness. By session 2, the voices were more positive and stable. At session 4, Mr. Smith noticed that the voices spoke less and at times no utterances were heard. By session 5, his family had seen changes: he was calmer, talked less alone, and was less invaded by his “scenes.” By the last session, *The Judge’s* voice had ceased. There was a clear diminishment of the invading psychotic symptoms. Throughout AT, Mr. Smith had a relatively high sense of presence (75%) with the avatar as measured with a small post-therapy questionnaire. While *The Judge’s* voice was gone, unfortunately the PSYRATS was unable to capture this clinical amelioration as Mr. Smith still had several voices. Objective improvements were observed following AT (Table 3). There were reductions of 24% on the total PANSS, 27, 29, and 20% on the positive, negative, and general symptomatology subscales of the PANSS, respectively as well as 75% on depressive symptoms. These improvements remained stable at our 3-month follow-up.

### Impressions of the Patient, Family, and Treating Psychiatrist

During interviews conducted with Mr. Smith, his family, and his treating psychiatrist (PL) 4 months succeeding the end of AT, they were all grateful with the outcome. Mr. Smith enjoyed AT as it led to the departure of his most distressing voice and he was able to learn new coping mechanisms. For his psychiatrist, Mr. Smith was amid his most severe cases. PL has vast experience for this complex population having treated over thousands of patients throughout his career. After AT, he noticed great changes in Mr. Smith. The frequency of his voices and delusions as well as his alcohol and cigarette intake reduced. He mostly noticed a beneficial change in the morale of the entire family. For the parents, their son was now more present both psychologically and physically. His mother reported noticing a substantial improvement in many areas of his life (i.e., quality of conversations, read the newspaper, and watch television). These changes were even noted by friends of the family.

## Discussion

In this reported case, a wide range of interventions to alleviate AVH had been offered to Mr. Smith. To our knowledge, he tried almost every possible treatment. It was apparent that he had been suffering from the persistency of all his voices for almost 20 years. He was hospitalized eight times and attempted treatments with little efficacy. Remarkably, several antipsychotic trials and nearly 70 rTMS and ECT sessions were put forward. Nothing ceased his distress to the extent that he attempted suicide on eight occasions and was aggressive toward others. This violence according to him was due to his unceasing voices. This case highlights the difficulty of finding an adequate treatment for ultra-resistant patients who remain symptomatic. While his treatment options were shortening, his treating psychiatrist (PL) jumped to the occasion to let him try AT, a new immersive and dialogical intervention.

Although he followed CBTp first, no symptomatic changes occurred. One potential explanation for the relative lack of efficacy may be that patients are not in direct relation with their voices. Typically, patients must imagine their persecutor and report the content of the voices to their therapist. CBTp is not built to elicit strong emotions and teach patients how to manage them as AT allows.

During AT, Mr. Smith improved in a way that was not observed with any other intervention and these improvements remained afterward. Mr. Smith had a satisfactory feeling of presence during AT. This is an important prerequisite for VR therapies (27), which enabled him to experiment more meaningful emotions to better regulate them through the relationship with his persecutor. With AT, Mr. Smith learned to control his anger and his symptom-related hostility reduced. Likewise, the therapy had an impact on the severity of his positive and disorganized symptoms as well as his depressive symptoms. Furthermore, *The Judge’s* voice disappeared, which helped his regain his life. The effects of AT went beyond the patient. The entire family benefited from AT as Mr. Smith was more physically and psychologically present and able to take part in familial activities. The interaction that Mr. Smith had with his avatar, *The Judge*, during AT gave him necessary skills and coping strategies that he was able to transfer more adequately into his daily life. Mr. Smith and his family attribute a lot of the improvements in his life to AT.

Although the results are beneficial, the primary measure of hallucinations was unable to showcase the benefits that were reported by the patient and his entourage. While the PSYRATS is widely used, this scale suffers from limitations for single clinical cases (23, 28). For instance, scales that evaluate each voice separately would have been better for the study of multiple voice hearers. Furthermore, to study the effects of the therapies, more baseline measures could have been measured to better delineate the psychiatric symptoms. However, prior to AT, we previously found that the symptoms of our patients were stable with multiple evaluations, which was probably the case for this patient (22). Finally, a longer follow-up period would have been interesting to ensure that these findings are maintained in time.

## Conclusion

This ultra-resistant case suggests that AT may be a useful intervention for refractory AVH in schizophrenia, even in clinical cases, where most therapeutic alternatives have been tried. Such improvements follow the general tendency seen in many other TRS patients after AT. The supposed benefits of AT seem above those of other psychotherapeutic treatment alternatives, which provide at best moderate benefits (29).

## Ethics Statement

The trial was approved by the ethics committee of the Institut Philippe-Pinel de Montréal. After discussion with a research nurse, Mr. Smith signed a detailed consent form. We obtained informed written consent from the patient authorizing publication of this clinical case.

## Author Contributions

LD and KP collected data on the patient. LD, SP, and AD wrote the paper. All authors provided critical comments. All authors approved the final version of the manuscript.

## Conflict of Interest Statement

AD and SP are holders of a grant from Otsuka Pharmaceuticals. The remaining coauthors declare that the research was conducted in the absence of any commercial or financial relationships that could be construed as a potential conflict of interest.

## Footnotes

^1^A pseudonym has been used to refer to the patient.
